# Of Fur, Feather, and Fin: Human’s Use and Concern for Non-Human Species

**DOI:** 10.3390/ani7030022

**Published:** 2017-03-09

**Authors:** Elizabeth Byrd, Nicole Olynk Widmar, Joan Fulton

**Affiliations:** Department of Agricultural Economics, Purdue University, West Lafayette, IN 47907, USA; byrd12@purdue.edu (E.B.); fultonj@purdue.edu (J.F.)

**Keywords:** animal use, animal welfare, livestock, pets, perceptions

## Abstract

**Simple Summary:**

An online survey of 825 U.S. residents was conducted to determine the level of approval for several uses of animals. More than 90% of respondents reported that using animals for egg production, service or therapy, pets, and milk production was acceptable to them. Older and male respondents more frequently found most of the animal uses surveyed acceptable. Half of respondents reported concern for the welfare of bison while 40% or more stated concern for the welfare of elk, beef cattle, and dairy cattle. Reported concern for animal species and acceptance of the use of animals were related in some instances. Respondents who stated they were concerned for the welfare of dairy cattle less frequently reported accepting using animals for meat production, livestock shows, and hunting.

**Abstract:**

The public’s concern for animal welfare is evolving and it is important to consider factors that are related to concern for animals and their use by humans. An online survey of 825 U.S. residents was conducted. Relationships between approval of animal uses and stated concern for animal welfare were examined. More than 90% of respondents reported that using animals for egg production, service or therapy, pets, and milk production was acceptable to them. Respondents who were younger or reported being female less frequently found most uses acceptable than older or male respondents. Half of respondents reported concern for the welfare of bison while 40% or more stated concern for the welfare of elk, beef cattle, and dairy cattle. Respondents who stated they were concerned for the welfare of dairy cattle less frequently reported accepting using animals for meat production, livestock shows, and hunting. Thus, self-reported concern for animal species and acceptance of the use of animals were related in some instances. A better understanding of the factors related to acceptance of animal uses and concern for animal welfare will help animal-related industries and wildlife agencies develop practices that are consistent with public attitudes.

## 1. Introduction

Controversy and public debate surrounding the use of animals by humans and concern about the welfare of those animals has long existed in the U.S. For example, the 1980s saw the founding of the People for the Ethical Treatment of Animals (PETA), increased regulation of animal use in research, and conflicts between businesses and animals, like fishing for tuna versus the safety of dolphins [1]. Concern for animal welfare has carried over to the concern for livestock animal welfare displayed in the 2000s. Through 2014, nine states including California, Florida, and Arizona passed bills or ballot initiatives to ban the use of gestation crates in swine production [2]. Likewise, McDonald’s, Burger King, and Smithfield Foods committed to pork raised without gestation crates [2]. Recently, Kroger announced that by 2025 it will have 100% of the eggs it sells produced without the use of battery cages [3]. The concern for animal welfare has expanded to wild animals with a Humane Society of the United States (HSUS) backed ballot initiative proposed to stop the use of dogs, traps, and bait in Maine’s recreational black bear hunts [4].

Animal use for human entertainment purposes has also been recently revisited. SeaWorld announced it would increase the size of its orca habitats in response to the backlash it faced following the release of the documentary “*Blackfish*” [5]. Just two years later, SeaWorld announced it would no longer breed orcas, it would not replace the orcas currently in its care, and it would phase out shows featuring orcas [6]. In 2016, Ringling Brothers and Barnum & Bailey Circus elephants performed for the last time after the show had featured an elephant act for 145 years [7]. Critics of elephant acts in the circus argue that the elephant shows belong in a different age. In fact, many cities have passed ordinances against such shows [8].

The public’s perception of acceptable animal uses is evolving and it is important to consider the factors that are related to or predispose members of the public to find using animals acceptable, to be concerned for animal welfare, and for their concern for animal welfare to evolve and change. Past studies linked being female and being a pet owner to increased concern for animals used in research [1]. Previous research also found respondents’ dietary choices and ethical motivations to be related to attitudes towards animals. Jabs, Devine, and Sobal [9] found that ethical concerns are one major reason participants adopted a vegetarian diet. Individuals who had adopted a vegetarian diet for ethical reasons were influenced by an animal-meat connection (connecting meat to the animal it came from) and animal welfare information [9]. Likewise, McKendree, Croney, and Widmar [10] found that respondents who had a source for animal welfare information more often reported concern for the welfare of domestic animals. 

Pet ownership was identified as a key factor associated with the concern for animal welfare in general [11]. Beyond pets, the public can also come into contact with animals through activities such as visiting a farm or fair where animals are being raised or exhibited and these visits could be related to attitudes towards animals. Cummins et al. [12] posited that an underlying tenant of agritourism is that showing the public what occurs during food animal production will lessen visitors’ concerns about animal welfare. County and state fairs are common across the U.S. and often feature livestock displays and shows where the public can see and sometimes interact with various kinds of livestock. Larger fairs also often have other animal related activities such as pony rides, petting zoos, circuses, and wagon rides.

In addition to individual respondent demographics, several studies have discussed the effect of the animal’s characteristics on respondent attitudes. For example, animals that are perceived to be rare (or valuable in some way), beautiful (or aesthetically pleasing), and admirable are seen more favorably, as are animals that are perceived to be useful (as opposed to those that are perceived to be detrimental) [13]. Previous research found the approval of an activity or use of animals is often dependent on the type of animal used [1]. Similarly, Serpell [13] points out that certain animal attributes such as being “cute”, “beautiful”, or “admirable” influence people’s emotional response to animals. It is also important to note that respondents were concerned about animals being exploited even when they were not being killed [14]. 

Acceptance of the human use of animals in various ways is fundamental and critical to understand before the nuances of livestock animal production systems or aspects of such uses can be evaluated. This research sought to determine the level of approval for 14 animal uses—namely, raised for meat, pets, hunting, horse racing, greyhound (dog) racing, livestock shows (i.e., county or state fair), horse-drawn carriages, service or therapy animals, zoo animals, circus animals, produce eggs, produce milk, petting zoos, and police/military dogs. The current research also explores the demographic factors related to approval or disapproval of animal uses, the level of concern for a number of wild and farmed animal species, and the interrelationship between approval of animal uses and stated concern for animal welfare.

## 2. Methods

### 2.1. Participants and Procedure

The data was collected via online survey administered in November of 2014 to a sample of 825 U.S. residents. The survey was administered using the Qualtrics platform. Lightspeed GMI, a provider that maintains a large opt-in panel of respondents, was used to recruit and contact potential survey respondents. The sample was targeted to be representative of the U.S. population in terms of gender, age, household income, education level, and region of residence [15]. Respondents were required to be at least 18 years of age to participate in the survey. The survey was approved by the local university’s institutional review board (IRB Protocol Number 1410015306).

### 2.2. Questionnaire

In addition to gender, age, household income, education level, and region of residence, information about pet (dog and/or cat) ownership, attitudes towards animal uses, and concern for animal welfare of various species were collected. It was also posited that other animal centered activities may be related to attitudes towards animal uses and animal welfare. Respondents were asked about their membership in animal welfare organizations such as PETA and the HSUS, and whether or not they had visited a livestock operation or fair. Specifically, respondents were asked “When was the last time you visited an operation of the following type?” The answer choices were “I have never visited such an operation”, “over 5 years ago”, and “less than 5 years ago”. The types of operations included were dairy farm, pig farm, horse farm, deer farm, state or county fair, and beef farm. For the purposes of this analysis, dairy, pig, and beef farms were aggregated to represent having visited a livestock operation. Furthermore, the categories of less than and over 5 years ago were aggregated to represent having visited such an operation.

Respondents answered a series of questions regarding their attitudes about animal uses and the welfare of animals. Respondents were asked “Do you find the following uses/roles/jobs for animals to be acceptable or unacceptable?” The list of uses included: raised for meat, pets, hunting, horse racing, greyhound (dog) racing, livestock shows (i.e., county or state fair), horse-drawn carriages, service or therapy animals, zoo animals, circus animals, produce eggs, produce milk, petting zoos, and police or military dogs. The potential responses were either acceptable or unacceptable. 

Respondents were asked to respond to the statement “Please indicate your level of concern regarding the welfare of the following animals” on a scale from one (extremely concerned) to five (not being concerned at all). The animal species respondents were asked about included deer, bison or buffalo, elk, feral pigs, beef cattle, dairy cattle, farmed pigs, chickens, wild turkey, catfish, and farmed turkeys.

### 2.3. Analysis

Cross tabulations were used to examine relationships between demographics, attitudes towards animal uses, and concern for the welfare of certain animal species. Cross tabulations have been used in similar analyses to explore the relationship between pet ownership and attitudes towards animal consumption [10] and the relationships between demographics and concern for animal welfare [11]. Statistically significant differences amongst responses were found using SPSS (IBM Corp., Armonk, NY, USA) based on z-scores. Pearson correlations were estimated using SAS (SAS Institute Inc., Cary, NC, USA) for select demographics to further explore the relationships between finding animal uses acceptable and concern for the welfare of animal species. A correlation between a continuous variable and a categorical variable with two categories is a point-biserial correlation which is equivalent to a Pearson correlation [16].

A logit model was used to determine how demographics impacted the acceptance of select animal uses. Using the question “Do you find the following uses/roles/jobs for animals to be acceptable or unacceptable?” a binary dependent variable (1 = acceptable, 0 = unacceptable) was constructed for this question. Logit models utilize maximum likelihood estimation. For a logit, the probability of a respondent finding an animal use acceptable (dependent variable = 1), is represented by,

(1) $$Pr_{i}(y=1|X_{i})=\frac{e^{\beta X_{i}}}{1+e^{\beta X_{i}}}$$

where *i* is the individual respondent and $X_{i}$ is a vector of variables and β is a vector of coefficients [17]. The sign of each estimated coefficient predicts the direction of a marginal change in a variable. The maximum likelihood logit model parameters and marginal effects were estimated using STATA 13 (Stata Corp, College Station, TX, USA).

## 3. Results

### 3.1. Demographics and Relationships with Animals

A total of 825 respondents completed the online survey which elicited demographics and information about relationships with animals. Demographics and summary statistics are shown in Table 1. Fifty-one percent of respondents were female and a total of 14% of respondents reported they were from 18 to 24 years old. Overall, 34% of respondents reported they fell into each of the 25 to 44 years of age and 45 to 64 years of age categories. Finally, 18% of respondents reported being 65 or older. Overall, 97% of respondents earned a high school diploma and 33% reported holding at least a bachelor’s degree. Six percent of respondents reported being vegetarian. A total of 65% of households reported owning a pet (dog or cat). Specifically, 49% owned at least one dog and 36% reported owning at least one cat. A total of 53% of respondents reported having visited a livestock operation (beef, dairy, or hog) in their lifetime and 67% reported having visited a fair.

### 3.2. Attitudes Towards Animal Uses

Respondents’ attitudes towards a series of uses/roles/jobs for animals are reported in Table 2. The highest rate of acceptance was for using animals for egg production with 93% of respondents finding it acceptable. A total of 92% of respondents found service or therapy animals, pets, and using animals to produce milk acceptable. Likewise, 88% of respondents approved of raising animals for meat and using dogs in police/military. A total of 83% of respondents approved animals being used in livestock shows, while 79% approved of petting zoos, 77% approved of horse-drawn carriages, and 76% approved of zoo animals. A total of 66% approved of horse racing and 63% approved of hunting. Finally, 52% of respondents found circus animals and greyhound racing acceptable.

Cross tabulations between finding an animal use acceptable and demographics are shown in Table 3. Several demographics, such as income, were also investigated; the results were not statistically significant and were omitted. Respondents over the age of 45 more frequently reported accepting most uses surveyed with the exception of hunting, dog racing, zoo animals, and circus animals where no significant differences between the age groups were found. Female respondents less frequently reported accepting hunting, horse racing, dog racing, and circus animals. In general, residents of the Northeast less often found hunting, horse-drawn carriages, zoo animals, egg production, and milk production acceptable animal uses. College graduates more frequently report accepting meat and hunting than those who did not graduate college. Non-vegetarians more frequently report accepting most uses surveyed except for horse racing, dog racing, and circus animals where no statistical differences were observed.

Cross tabulations between finding an animal use acceptable and animal involvement are shown in Table 4. Pet owners more frequently reported acceptance of using animals for pets and therapy animals. On the other hand, pet owners less frequently reported accepting horse racing and circus animals. Members of HSUS/PETA less frequently reported accepting meat, egg and milk production, petting zoos, police/military animals, hunting, horse and dog racing, zoo animals, and circus animals. Respondents who had visited a livestock operation more frequently reported acceptance of raising animals for meat, livestock shows, petting zoos, police/military animals, hunting, horse racing, dog racing, horse-drawn carriage, and zoo animals. Those respondents who reported having visited a fair more frequently reported accepting animal uses except for horse racing, dog racing, zoo animals, and circus animals than those who have not visited a fair.

Results of the logit analysis relating demographics to acceptance of six animal uses are shown in Table 5. For “accepting uses: animal for meat production”, being under 45, vegetarian, or a member of HSUS/PETA were associated with a 4, 36, and 9% decrease in the probability of reporting accepting utilizing animals for meat production, respectively. The results are similar for individuals accepting animal use for egg production and milk production. However, in the case of egg production, pet ownership and having visited a fair each result in a 4% increase in the probability of accepting animals be used for egg production. In the case of milk production, having visited a livestock operation is associated with a 3% decrease in the probability of accepting milk production as an animal use. However, being a college graduate, a pet owner, or having visited a fair increase the probability of accepting milk production by 4, 2, and 6% respectively. 

Three animal uses that include the use of dogs (pets, police animals, dog racing) were also studied (Table 5). Being under 45 and being a vegetarian were associated with 4% and 12% decrease in the probability of finding pets an acceptable animal use. However, owning a pet increased the probability by 5% and visiting a fair increased the probability by 8%. The results were similar with accepting police animals. In the case of dog racing, being a female decreased the probability of accepting it by 11% and being a pet owner resulted in a 7% decrease in the probability of accepting dog racing as a use of animals.

### 3.3. Concern for Animal Welfare

Respondents were asked whether or not they were concerned for the welfare of 10 animal species, both farmed and wild (Table 2). Half of respondents reported they were concerned for the welfare of bison or buffalo and 43% of respondents were concerned for the welfare of elk. A total of 41% of respondents reported being concerned for the welfare of beef cattle while 40% expressed concern for dairy cattle. A total of 38% of respondents reported being concerned for the welfare of deer and chickens and 37% were concerned for the welfare of farmed pigs. A total of 33% of respondents reported being concerned for the welfare of farmed turkeys while 31% stated concern for the wild turkey. Feral pigs and catfish received the lowest levels of concern with 24% of respondents stating they were concerned for the welfare of these species. Overall, the average level of concern across all species was 3.04 with a standard deviation of 0.97 (Table 2).

Cross tabulations between demographics and concern for animal species were examined (Table 6). Respondents over 45 more frequently indicated concern for bison, but those under 45 more frequently reported concern for the feral pig. Female respondents more frequently reported being concerned for the welfare of beef cattle, dairy cattle, farmed pigs, chickens, and farmed turkeys than did male respondents. Interestingly, all the species for which females reported concern statistically more frequently than men were domestic food animals. Residents of the Northeast more frequently reported being concerned for the welfare of bison, feral pigs, and farmed pigs. College graduates more frequently reported being concerned for the welfare of farmed turkeys than those who had not graduated college. Finally, those respondents who identified themselves as being either vegetarian or members of HSUS and/or PETA more frequently reported being concerned for the welfare of all animals studied.

Cross tabulations between acceptance of animal uses and concern for different species are presented in Table 7 (wild species) and Table 8 (farmed species). In particular, respondents who report not being concerned for the welfare of the wild species included in the survey more frequently report raising meat an acceptable animal use. Considering farmed animal welfare, those respondents who reported not being concerned for the welfare of farmed animal species (beef cattle, dairy cattle, farmed pigs, chickens, and farmed turkey) more frequently reported finding raising animals for meat, hunting, horse racing, dog racing, horse-drawn carriage, zoo animals, and circus animals acceptable uses of animals. Of those respondents that reported accepting egg production as an animal use, respondents more often reported not being concerned with the welfare of chickens. Similarly, of those respondents who report accepting livestock shows, respondents more frequently report not being concerned for the welfare of dairy cattle, farmed pigs, chickens, and farmed turkeys. 

## 4. Discussion

### 4.1. Demographics and Relationships with Animals

This sample was more educated than the national average where 85.7% of Americans over 25 years of age have graduated high school and 28.5% of have at least a four-year degree [15]. The education level of these survey respondents was similar to other online surveys [11,12]. Past internet surveys have overrepresented those who were more highly educated [18,19], likely because more educated residents were more likely to have internet access. Six percent of respondents reported being vegetarian and 3% of respondents reported that they were vegan, which is consistent with a previous nationwide survey in which 4% of respondents identified themselves as vegetarian and 2% as vegan [11]. In addition to dietary habits, an individual’s association with animals in various settings had been hypothesized to influence their stated concern for animal welfare. For example, pet ownership is a key factor related to general animal welfare concern [11]. The public could also come into contact with animals by visiting a fair (e.g., state or county) or visiting a livestock operation. Thus, it is possible those individuals who have visited farms or fairs may be more accepting of uses of animals such as raising for meat, milk, and eggs or livestock shows.

### 4.2. Acceptance of Animal Uses

In the current study, animal use for food, even for meat where the animal must be slaughtered, had a high acceptance rate. This is consistent with previous findings that a majority of survey respondents are not opposed to eating beef cows, dairy cows, pigs, chickens, turkeys, or milk/dairy products [11]. Interestingly, Wells and Hepper [14] found that respondents expressed more concern for uses that lead to the animal’s death or injury (dog-fighting, medical research) than for uses that were exploitative (dog racing, horse racing, circuses, and zoos). However, Knight et al. [20] found that respondents approved of using animals for experimentation and in classrooms more than they approved of animal use for personal decoration, entertainment, or financial gain. It can be argued that experimentation and classroom education are perceived as beneficial to people, whereas uses such as entertainment could be viewed as frivolous. This is certainly consistent with the current survey results where greyhound (dog) racing and circuses, which could be understood as being entertainment, were the least widely accepted uses with 52% of respondents approving of these uses. Likewise, uses such as pets, service/therapy animals, and police/military dogs could be seen as beneficial to people much like experimentation or educational purposes. The idea that animals can be used in ways that are beneficial to people also aids in explaining why food uses food production (e.g., meat, milk, eggs) are so widely accepted. Although this begs the question, do people approve of using animals for food production because the survey takers are consumers of these products (only 6% identified themselves as vegetarian) or do people consume these products because they believe animals can be used for beneficial uses such as providing food?

It is hypothesized that animal use for food (meat, milk, and/or dairy) is more acceptable (or acceptable to a larger proportion of people) than animal use for activities that are viewed as exploiting animals for non-food reasons such as entertainment or personal decoration. Greyhound racing is an example of an activity that respondents may view as a non-food exploitative of animals. The low acceptance rate for greyhound racing amongst U.S. residents is understandable given that 39 states have laws prohibiting commercial dog racing and there are only five states with active greyhound racing tracks [21]. 

Recently, the use of elephants in the circus has been in the news with Ringling Bros. and Barnum & Bailey Circus ending elephant shows and retiring the elephants. This low acceptance of circus animals is consistent with the discussion of Wells and Hepper [14] who point out that participants in their survey were concerned about the use of circus animals even though the animals were being “exploited” rather than killed or injured. An interesting contrast to circus animals, many of which are wild animals, is the acceptance of zoo animals. The general public sees conservation as the zoo’s main role [22]. Thus, it is likely the public does not view zoos as inherently exploitative and approves of them at a higher rate than circuses. Interestingly, zoo visitors have been found to perceive the animals in zoos as happier and less bored than non-zoo goers [22], which could help explain why zoos are not more universally considered an acceptable animal use.

The results of the current study indicate varying levels of support for different uses of the same animal species. For example, 92% of respondents approved of animals as pets or service animals, similar high support for police/military dogs, and low support (52%) for dog racing. Serpell [13] points out that certain animal attributes such as animals that are aesthetically pleasing (e.g., cute or beautiful) influence people’s emotional response to animals. People certainly have emotional responses to their own pets and likely to animals perceived to be pets. McKendree et al. [11] found that the animals most survey respondents were opposed to eating were also those classified most often as pets. Pets are universally regarded highly and uses that are seen as exploiting animals commonly believed to be pets, such as dog racing, would likely receive less approval. For example, in the current study more people approve of pets and therapy animals than dog racing.

Several demographic factors are related to attitudes towards animals and their uses. Respondents over the age of 45 more frequently reported finding many of the uses acceptable than their younger counterparts, consistent with McKendree et al. [10] who found that younger respondents were more likely to report concern for animal welfare. The logit model revealed being under 45 had statistically significant decreases in the probability of approving of five of the six uses tested. Being under 45 accounted for a 4% to 8% decrease in the probability of approving of animal uses with eggs, milk, and police animals seeing the largest decreases. Likewise, female respondents less frequently reported accepting hunting, horse racing, dog racing, and circus animals, consistent with previous research in which women more frequently report concern for medical research animals [23] and farm animals [10]. This result has also been found in other countries; Phillips et al. [24] found that women in 11 Eurasian countries were more concerned about animal welfare than their male counterparts. Furthermore, Wells and Hepper [14] found that women disagreed more than men with deer hunting, dog racing, horse racing, and using animals in the circus. The logit results revealed being female resulted in 10% decrease in the probability of approving of dog racing. However, no other significant impact was seen for the other uses modeled. It is possible other factors are becoming stronger indicators of the approval of animal uses than gender.

Residents of the Northeast less frequently reported accepting several animal uses including horse-drawn carriages, zoo animals, egg production, milk production, and hunting. Specific to livestock production, it may also be that the Northeast is further removed from food production. According the American Egg Board [25], the Northeast has no states which rank in the top 10 in egg production. Similarly, the Northeast region, as defined by the U.S. Census, contains only 15% of the U.S. dairy cow herd [26]. Likewise, horse-drawn carriages in New York City’s Central Park have been in the news following public and political outcries for a ban [27]. Thus, it is probable that the Northeast has seen more media attention with regard to horse-drawn carriages. Likewise, the Northeast region participates in hunting at a rate lower than the national participation rate of 6% [28]. 

Not surprisingly, those respondents who did not identify as vegetarian more frequently found most of the animal uses surveyed acceptable. Similarly, reporting oneself as a vegetarian resulted in a 0.36 decrease in the probability of approving of using animals for meat. Jabs, Devine, and Sobal [9] found that a major reason respondents adopted a vegetarian diet was due to ethical concerns. They found individuals who had adopted a vegetarian diet for ethical reasons were influenced by an animal-meat connection (connecting meat to the animal it came from) and animal welfare information [9]. Thus, it is not surprising that vegetarians are less approving of a variety of animal uses. However, some uses were more universally unacceptable; statistically significant differences between vegetarians and non-vegetarians were not found for horse racing, dog racing, and circus animals. 

It is not surprising that pet owners would be accepting of their own actions—namely keeping pets. Past studies linked being a pet owner to increased concern for animals used in various types of research [1]. More recently, pet ownership was identified as a key factor associated with the concern for animal welfare in general [11]. Wells and Hepper [14] pointed out that respondents were also concerned about uses they felt exploited animals even when they were not being killed. In the current research, pet owners were less accepting of horse racing and circus animals which could be perceived to be exploiting animals. On the other hand, pet owners were more accepting of uses such as pets and therapy animals, which could mean these are not seen as exploitative. 

The finding that members of two popular animal welfare organizations less frequently accept the animal uses studied is not surprising. These results were confirmed by logit analysis where being a member of HSUS or PETA resulted in a decrease in the probability for the animal uses modeled except for pets. For example, PETA proclaims on its website “Animals are not ours to eat, wear, experiment on, use for entertainment or abuse in any way” [29]. Similarly, the HSUS lists their efforts to ban gestation crates and battery cages in farming, captive hunts, hunting of certain animals with dogs, and several other efforts [30]. The findings in the current research, namely that members of HSUS and/or PETA are less supportive of animal uses are consistent with previous research.

Respondents who reported visiting a livestock operation or a fair more frequently reported approving of most of the animal uses. Cross tabulations report proportions of the sample that answered questions with given responses, but cannot point to causation. Thus, it is possible that those who are more accepting of animal uses are more likely to seek out and visit farms and fairs. It is also possible that those who visit farms and/or fairs become more accepting of various animal uses because of their visit. Previous research found that those who had visited a livestock operation did not differ significantly in age, gender, or region of residency and those who had visited were more likely to have owned or were related to someone who operated a livestock operation [12]. This suggests that those who visited livestock operations are predisposed to accept various animal uses rather than the converse. Previous research points out that the general public tends to have a high level of concern for food animal welfare but low levels of knowledge and understanding of animal welfare [31]. It could also be argued that those who visit livestock operations could be more educated about animal welfare.

### 4.3. Concern for Animal Welfare

Finding that bison and elk were the animals respondents were most concerned for is consistent with the discussions in Driscoll [22] who summarized previous findings where activities involving large attractive mammals have been found to be less acceptable. Likewise, Serpell [13] found that animals that are rare, beautiful, and admirable are seen more favorably. These two wild species have a range that does not encompass the entire U.S. and could be seen as rare by respondents. A total of 38% of respondents reported being concerned for the welfare of deer. As Serpell [13] points out, they are often over-abundant and pose traffic hazards, but the public often protects them from lethal measures to control their population. It is not surprising that such a low percentage of respondents were concerned for the welfare of feral pigs, since animals perceived to be detrimental are often regarded less positively than beneficial animals [13]. 

Approximately the same percentage of respondents reported being concerned for the welfare of beef (41%) and dairy (40%) cattle. It is interesting to note the similar level of concern for these two livestock animals given that cattle must necessarily be slaughtered to produce beef, but cattle do not need to be slaughtered to produce milk. Being concerned about the welfare of farm animals does not necessarily equate to not consuming the products from them. In this case, few survey respondents reported being vegetarian or vegan, but a much higher percentage report being concerned for the welfare of food animal species.

Residents of the Northeast more frequently reported being concerned for the welfare of bison, feral pigs, and farmed pigs. Previous research found that residents of the Midwest were less concerned about pig production practices; in that study, the authors hypothesized that residents of the Midwest, which contains many top producing pork states, live in an area where pork farming is very important and are less concerned about the welfare of farmed pigs [10]. One potential explanation for the increased rates of concern amongst residents of the Northeast, is that unlike residents of the Midwest, they are removed from pork production. 

## 5. Conclusions 

It is important to understand people’s ever changing attitudes towards various animals and their uses. For example, such information can be used to generate public support for conservation efforts [23], understand the public’s opinion on regulations on animal use [32], and track how public opinion of animal uses and animal species is affected by science [13]. This research considered U.S. residents’ attitudes towards acceptable uses of animals. The public’s concern for animal welfare is evolving and it is important to consider factors that are related to or prompt consumers to be concerned for the welfare of animals in a variety of uses. Overall, more than 90% of respondents found using animals for egg production, service or therapy animals, pets, and milk production acceptable. On the other hand, only 52% of respondents agreed with dog racing or circus animals. The demographic factors related to approval or disapproval of animal uses was also explored. Respondents who were younger or reported being females less frequently found most uses acceptable than older or male respondents. Significant regional differences were found; residents of the Northeast less frequently reported accepting horse-drawn carriages, zoo animals, egg production, milk production, and hunting. The level of concern for a number of wild and farmed species was also explored. Half of respondents were concerned for the welfare of bison while 40% or more stated concern for the welfare of elk, beef cattle, and dairy cattle. Finally, the relationship between the approval of animal uses and the concern for animal welfare was examined. Considering farmed animal welfare, those respondents who reported not being concerned for the welfare of farmed animal species more frequently reported finding raising animals for meat, hunting, horse racing, dog racing, horse-drawn carriage, zoo animals, and circus animals acceptable uses of animals. Thus, relationships were found between expressing concern for the welfare of a species generally and the use of that species (or other animal species) for different purposes.

While this study yields interesting results, it is important to consider its limitations. This research relied on cross tabulations and logit analysis. Several non-standard measurements of constructs were also employed. Future research could focus on other statistical/econometric tools (e.g., *t*-tests, ANOVA, additional Pearson correlations) and standard measurements (e.g., Likert scaling). Future authors might consider a broader list of animal species and animal uses to explore and including multiple questions to address each construct to avoid the question of reliability of single items. 

Future research may aim to help understand the public acceptance of animal uses and be aware of shifts in public perception. While understanding public perception and acceptance of specific practices is important, understanding the underlying values the public holds for animals is equally important. A better understanding of the factors related to attitudes towards individual animal uses and concern for animal welfare will help animal related industries and wildlife agencies develop practices that are consistent with public and consumer attitudes.

## Figures and Tables

**Table 1:** Survey demographics.

Demographic Variable	Value
Male	49%
Age	
18 to 24 years	14%
25 to 44 years	34%
45 to 64 years	34%
65 years and over	18%
Annual Pre-Tax Household Income	
Less than $20,000	19%
$20,000–39,999	29%
$40,000–59,999	23%
$60,000–79,999	12%
$80,000–99,999	7%
$100,000–119,999	3%
Education	
Did not graduate from high school	3%
Graduated from high school, did not attend college	22%
Attended college, no degree earned	26%
Attended college, Associates or Trade degree	15%
Attended college, Bachelor’s degree earned	23%
Graduate or advanced Degree (M.S., PhD., J.D)	10%
Region of Residence	
Northeast	17%
South	33%
Midwest	27%
West	23%
Other Demographics of Interest	
Respondent is a member of HSUS/PETA	7%
Respondent is vegetarian	6%
Pet owner (e.g., owns at least one dog or cat)	65%
Have Visited a Livestock Operation (beef, dairy, hog)	53%
Have Visited a Fair (state or county)	67%

**Table 2:** Responses to finding animal uses acceptable.

**Found the Following Uses/Roles/Jobs for Animals Acceptable**	
Produce eggs	93%
Pets	92%
Produce milk	92%
Service or therapy animals	92%
Raised for meat	88%
Police or military dogs	88%
Livestock shows (i.e., state or county fair)	83%
Petting zoos	79%
Horse-drawn carriages	77%
Zoo animals	76%
Horse racing	66%
Hunting	63%
Greyhound (dog) racing	52%
Circus animals	52%
**Concern for animal welfare of the following species**	
Bison	50%
Elk	43%
Beef cattle	41%
Dairy cattle	40%
Deer	38%
Chickens	38%
Farmed pigs	37%
Farmed turkey	33%
Wild turkey	31%
Feral pigs	24%
Catfish	24%

Notes: Respondents were asked, “Do you find the following uses/roles/jobs for animals to be acceptable or unacceptable?” For the purposes of this table, respondents who answered (1) extremely concerned and (2) relatively concerned were aggregated into a single category of being concerned about the species in question.

**Table 3:** Cross tabulations between acceptable animal uses and demographics.

	Animal Use	Age		Gender		Region				Education		Vegetarian	
		Over 45	Under 45	Female	Male	Midwest (a)	South (b)	West (c)	Northeast (d)	Did Not Graduate College	College Graduate	No	Yes
**Finds Use Acceptable**	Meat	**91.2**	**85.5**	86.9	90.1	86.2	89.4	91.1	86.5	**84.5**	**89.8**	**90.5**	**55.3**
	Produce eggs	**96.5**	**88.3**	91.3	92.1	**94.0 bd**	**95.6 cd**	**90.1 b**	**87.9 ab**	90.3	93.4	**93.3**	**80.9**
	Produce milk	**96.8**	**86.7**	92.6	91.4	92.7	**94.5 d**	90.1	**88.7 b**	89.3	92.9	**93.1**	**74.5**
	Livestock shows	**89.4**	**75.0**	81.9	83.3	82.6	84.7	80.2	81.6	81.1	83.0	**83.7**	**63.8**
	Pets	**94.0**	**88.8**	91.9	91.1	89.4	93.8	91.7	90.1	89.8	92.1	**92.3**	**78.7**
	Petting zoo	**82.9**	**73.7**	77.8	79.3	**75.7 b**	**82.8 a**	**77.6**	**75.9**	79.6	78.2	**79.7**	**59.6**
	Police animals	**92.8**	**83.2**	89.3	87.2	87.2	91.2	85.9	87.2	87.4	88.5	**89.1**	**74.5**
	Therapy animals	**94.7**	**88.0**	92.6	90.4	92.2	**93.8 c**	**88.5 b**	90.1	88.3	92.6	**92.2**	**80.9**
	Hunting	62.4	63.5	**56.1**	**70.0**	**65.1 d**	**65.3 d**	**64.6 d**	**52.5 abc**	**55.8**	**65.3**	**63.9**	**46.8**
	Horse racing	**71.8**	**59.2**	**61.1**	**70.7**	63.3	68.6	66.1	63.8	63.6	66.6	66.6	53.2
	Dog racing	51.7	51.3	**45.6**	**57.6**	51.4	55.5	48.8	48.2	51.5	51.5	52.2	40.4
	Horse-drawn carriage	**83.1**	**70.2**	74.5	79.6	**76.6 d**	**80.3 d**	**80.2 d**	**66.7 abc**	72.8	78.4	**78.3**	**55.3**
	Zoo animals	72.8	78.3	78.3	72.8	**77.5 d**	**79.9 d**	73.4	**66.7 ab**	73.3	76.3	**76.5**	**59.6**
	Circus animals	54.3	50.3	**46.1**	**58.9**	52.8	**56.9 c**	**46.9 b**	50.4	54.4	51.7	52.7	46.8

Note 1: In the interest of brevity, the values representing the percentage of individuals in each category who found the animal use unacceptable has been omitted. Because the values in each column must necessarily total 100, omitted variables can be calculated. Note 2: The numbers appear in bold when there was a statistically significant difference at the 5% level. For region, the letters in each column represent statistically significant differences amongst the different regions.

**Table 4:** Cross tabulations between acceptable animal uses and animal involvement.

	Animal Use	Pet Ownership		Member of HSUS/PETA		Visited a Livestock Operation		Visited a Fair	
		Non Pet Owner	Pet Owner	Not a Member	Member	Have Not Visited	Have Visited	Have Not Visited	Have Visited
**Finds Use Acceptable**	Meat	87.3	89.1	**89.1**	**80.3**	**84.1**	**92.4**	**82.9**	**91.3**
	Produce eggs	90.7	93.6	**93.2**	**85.2**	92.1	93.1	**88.4**	**94.7**
	Produce milk	91.1	92.5	**92.5**	**85.2**	91.6	92.4	**86.5**	**94.7**
	Livestock shows	83.8	81.8	83.2	73.8	**79.5**	**85.3**	**73.5**	**87.1**
	Pets	**88.0**	**93.4**	91.5	91.8	90.3	92.6	**85.8**	**94.4**
	Petting zoo	78.4	78.7	**79.5**	**67.2**	**75.7**	**81.1**	**74.5**	**80.5**
	Police animals	86.3	89.3	**89.0**	**78.7**	**86.7**	**89.6**	**80.0**	**92.4**
	Therapy animals	**88.7**	**93.1**	91.4	93.4	89.8	93.1	**84.7**	**94.9**
	Hunting	61.5	63.7	**63.9**	**50.8**	**56.0**	**69.1**	**53.1**	**67.8**
	Horse racing	**71.5**	**62.7**	**67.3**	**47.5**	**60.6**	**70.5**	63.3	67.1
	Dog racing	55.7	49.3	**52.6**	**37.7**	**46.5**	**57.2**	49.8	52.4
	Horse-drawn carriage	80.1	75.3	77.7	67.2	**72.1**	**81.3**	**68.4**	**81.3**
	Zoo animals	78.0	74.2	**76.4**	**63.9**	**75.4**	**75.6**	74.9	75.8
	Circus animals	**60.8**	**47.8**	**53.8**	**34.4**	52.4	52.3	53.5	51.8

Note 1: In the interest of brevity, the values representing the percentage of individuals in each category who found the animal use unacceptable has been omitted. Because the values in each column must necessarily total 100, omitted variables can be calculated. Note 2: The numbers appear in bold when there was a statistically significant difference at the 5% level.

**Table 5:** Summary of logistic regression analysis for variables predicting finding an animal use acceptable.

Variable	Respondent Finds the Following Animal Use Acceptable											
	Meat		Eggs		Milk		Pets		Police Animals		Dog Racing	
	Coefficient (SE)	Marginal Effect (SE)	Coefficient (SE)	Marginal Effect (SE)	Coefficient (SE)	Marginal Effect (SE)	Coefficient (SE)	Marginal Effect (SE)	Coefficient (SE)	Marginal Effect (SE)	Coefficient (SE)	Marginal Effect (SE)
*Under 45*	−0.4861 ** (0.2418)	**−0.0401** **(0.0199)**	−1.3656 *** (0.3233)	**−0.0727** **(0.0173)**	−1.5221 *** (0.3271)	**−0.0818** **(0.0177)**	−0.6409 ** (0.2744)	**−0.0417** **(0.0180)**	−0.8860 *** (0.2440)	**−0.0783** **(0.0216)**	0.0553 (0.1472)	0.0138 (0.0367)
*Female*	−0.3332 (0.2362)	−0.0.271 (0.0191)	0.0306 (0.2798)	0.0015 (0.0137)	0.0281 (0.2743)	0.0014 (0.0134)	0.0066 (0.2591)	0.0004 (0.01645)	0.1091 (0.2277)	0.0095 (0.0195)	−0.4419 *** (0.1484)	**−0.1099** **(0.0352)**
*College graduate*	0.6585 *** (0.2571)	0.0616 (0.0271)	0.7375 ** (0.3088)	**0.0432** **(0.0210)**	0.8234 *** (0.3028)	**0.0490** **(0.0213)**	0.4377 (0.2913)	0.0306 (0.0222)	0.2681 (0.2615)	0.0242 (0.0249)	−0.0412 (0.1668)	−0.0102 (0.0416)
*Vegetarian*	−2.222 *** (0.3455)	**−0.3639** **(0.0784)**	−1.046 ** (0.4306)	**−0.0786** **(0.0460)**	−1.4286 *** (0.4001)	**−0.1248** **(0.0543)**	−1.1717 *** (0.4040)	**−0.1168** **(0.0569)**	−0.8738 ** (0.3810)	**−0.1019** **(0.0573)**	−0.4701 (0.3146)	−0.1163 (0.0760)
*HSUS/PETA*	−0.8581 ** (3868)	**−0.0940** **(0.0540)**	−1.1187 *** (0.4301)	**−0.0853** **(0.0465)**	−0.9388 ** (0.4368)	**−0.0663** **(0.0420)**	−0.0957 (0.5169)	−0.0063 (0.0352)	−0.9841 *** (0.3654)	**−0.1176** **(0.0567)**	−0.5521 ** 0.2820	**−0.1362** **(0.0673)**
*Pet ownership*	0.2339 (2476)	0.01958 (0.0213)	0.7610 ** (0.2993)	**0.0419** **(0.081)**	0.5382 * (0.2959)	**0.0284** **(0.0168)**	0.7898 *** (0.2712)	**0.0562** **(0.0209)**	0.4716 * 0.2427	**0.0428** **(0.0232)**	−0.2927 * (0.0570)	**−0.0729** **(0.0381)**
*Visited a livestock operation*	0.7120 ** (0.2796)	**0.0594** **(0.0235)**	−0.5073 (0.3914)	−0.0248 (0.0166)	−0.6367 * (0.3365)	**−0.0310** **(0.0164)**	−0.4372 (0.3182)	−0.0276 (0.0198)	−0.3883 (0.2763)	−0.0330 (0.0233)	0.4909 *** (0.1656)	**0.1220** **(0.0409)**
*Visited a fair*	0.4029 (0.2630)	0.0347 (0.0239)	0.8565 * (0.3274)	**0.0486** **(0.0212)**	1.1176 *** (0.3219)	**0.0664** **(0.0227)**	1.054 *** (0.3078)	**0.0796** **(0.0264)**	1.1609 *** 0.2676	**0.1183** **(0.0309)**	−0.0646 (0.1733)	−0.0163 (0.0432)
*Constant*	1.6247 (0.3210)		2.335 (0.3993)		2.3994 0.3998		1.679 0.3504		1.672 (0.03221)		0.3323 0.2147	
*Log*	−258.89		−192.96		−196.97		−220.61		−270.7839		−556.33	
*Likelihood*												
*Prob > Chi2*	0.0000		0.0000		0.0000		0.0000		0.0000		0.0000	
*Pseudo R2*	0.1214		0.1131		0.1435		0.0794		0.0935		0.0265	
*n*	825		825		825		825		825		825	

Note 1: Statistical significance is denoted at the 10% *, 5% **, and 1% *** levels. Note 2: The numbers appear in bold when there was a statistically significant difference at the 5% level.

**Table 6:** Cross tabulations between demographics and concern for the welfare of wild and domestic animals.

	Concern for Animal Species	Age		Gender		Region				Education		Vegetarian		Member of HSUS/PETA	
		Over 45	Under 45	Female	Male	Midwest (a)	South (b)	West (c)	Northeast (d)	Did Not Graduate College	College Graduate	No	Yes	Not a Member	Member
**Wild**	Deer	38.6	37.5	39.1	36.9	33.9	36.5	40.1	44.7	41.3	37.0	**36.5**	**63.8**	**35.5**	**70.5**
	Bison	**54.0**	**45.9**	50.6	49.8	**44.0 d**	48.2	53.1	**59.6 a**	47.1	51.2	**48.6**	**76.6**	**47.9**	**78.7**
	Elk	44.6	40.3	45.3	39.7	38.5	40.9	44.3	49.6	41.3	43.0	**40.6**	**74.5**	**40.6**	**67.2**
	Feral pig	**18.7**	**29.8**	23.9	24.1	**16.5 d**	25.5	26.0	**29.8 a**	21.4	24.9	**22.0**	**57.4**	**22.1**	**47.5**
	Wild turkey	30.0	31.6	30.5	31.0	26.6	32.1	30.7	34.8	32.0	30.4	**28.9**	**61.7**	**29.2**	**30.4**
	Catfish	20.8	26.5	24.8	22.2	19.7	25.2	24.5	24.8	22.3	23.9	**22.1**	**46.8**	**22.3**	**39.3**
**Domestic**	Beef cattle	40.9	40.6	**45.1**	**36.2**	34.9	38.7	45.3	47.5	38.3	41.5	**39.1**	**68.1**	**38.7**	**65.6**
	Dairy cattle	40.9	38.5	**44.2**	**35.2**	33.9	37.2	45.8	45.4	37.4	40.5	**38.0**	**68.1**	**37.2**	**72.1**
	Farmed pigs	37.0	37.2	**43.0**	**31.0**	**30.3 d**	34.3	42.7	**45.4 a**	33.0	38.4	**35.3**	**66.0**	**34.8**	**65.6**
	Chicken	37.4	37.8	**43.0**	**32.0**	35.3	36.5	39.6	40.4	32.5	39.3	**36.8**	**51.1**	**36.0**	**57.4**
	Farmed turkey	33.0	33.9	**37.2**	**29.6**	30.3	31.4	36.5	38.3	**27.2**	**35.5**	**32.3**	**53.2**	**31.5**	**57.4**

Note 1: In the interest of brevity, the values representing the percentage of individuals in each category who found the animal use unacceptable has been omitted. Because the values in each column must necessarily total 100, omitted variables can be calculated. Note 2: The numbers appear in bold when there was a statistically significant difference at the 5% level. For region, each column the letters represent statistically significant differences amongst the different regions. Note 3: For the purposes of this table, (1) extremely concerned and (2) relatively concerned were aggregated into a single category being concerned about the species in question.

**Table 7:** Cross tabulations between concern for the welfare of wild animals and acceptance of animal uses.

	Animal Use	Concerned About the Welfare of											
		Deer		Bison		Elk		Feral Pig		Wild Turkey		Catfish	
		No	Yes	No	Yes	No	Yes	No	Yes	No	Yes	No	Yes
**Finds Use Acceptable**	Meat	**91.6**	**83.4**	**91.0**	**86.0**	**92.0**	**83.8**	**92.0**	**77.3**	**92.1**	**80.3**	**91.9**	**77.3**
	Produce eggs	93.9	90.4	92.5	92.8	93.5	91.5	**94.6**	**86.4**	**94.4**	**88.6**	**93.8**	**88.7**
	Produce milk	92.8	90.8	91.5	92.5	92.6	91.2	**93.5**	**87.4**	**93.7**	**88.2**	**93.5**	**87.1**
	Livestock shows	**85.5**	**77.7**	83.7	81.4	84.4	80.1	**85.6**	**72.7**	**84.9**	**77.2**	**84.9**	**74.7**
	Pets	90.4	93.3	**88.8**	**94.2**	90.5	92.9	92.0	89.9	92.1	90.2	91.6	91.2
	Petting zoo	79.8	76.4	80.5	76.6	80.0	76.6	**80.2**	**73.2**	79.9	75.6	78.9	77.3
	Police animals	88.3	88.2	87.6	88.9	88.8	87.5	**89.8**	**83.3**	**56.6**	**43.8**	88.7	86.6
	Therapy animals	91.8	91.1	**89.3**	**93.7**	90.1	93.4	92.2	89.4	92.3	89.8	91.4	91.8
	Hunting	**68.9**	**53.2**	**67.4**	**58.5**	**68.4**	**55.6**	**67.0**	**50.0**	**66.9**	**53.9**	**66.2**	**52.1**
	Horse racing	**71.6**	**56.4**	**70.1**	**61.6**	**70.0**	**60.1**	**69.4**	**54.5**	**70.2**	**55.9**	**68.9**	**55.7**
	Dog racing	**58.1**	**40.8**	**58.4**	**44.7**	**57.6**	**43.3**	53.3	46.0	**55.5**	**42.5**	**54.0**	**43.3**
	Horse-drawn carriage	**81.8**	**69.1**	**80.8**	**73.2**	**80.8**	**71.8**	**80.4**	**66.2**	**80.9**	**30.8**	**80.3**	**66.0**
	Zoo animals	**78.1**	**71.3**	**81.0**	**70.0**	77.8	72.4	**78.0**	**67.7**	**78.1**	**69.7**	76.7	71.6
	Circus animals	**56.8**	**45.2**	**60.3**	**44.4**	**58.4**	**44.2**	**55.3**	**42.9**	54.3	48.0	52.6	51.5

Note 1: In the interest of brevity, the values representing the percentage of individuals in each category who found the animal use unacceptable has been omitted. Because the values in each column must necessarily total 100, omitted variables can be calculated. Note 2: The numbers appear in bold when there was a statistically significant difference at the 5% level. Note 3: For the purposes of this table, (1) extremely concerned and (2) relatively concerned were aggregated into a single category being concerned about the species in question.

**Table 8:** Cross tabulations between concern for the welfare of farmed animals and acceptance of animal uses.

	Animal Use	Concerned About the Welfare of									
		Beef Cattle		Dairy Cattle		Farmed Pigs		Chicken		Farmed Turkey	
		No	Yes	No	Yes	No	Yes	No	Yes	No	Yes
**Finds Use Acceptable**	Meat	**92.6**	**82.4**	**93.0**	**81.7**	**92.5**	**81.7**	**91.5**	**83.5**	**91.4**	**82.6**
	Produce eggs	93.9	90.8	93.0	92.1	93.4	91.2	**94.4**	**89.7**	92.9	92.0
	Produce milk	93.0	90.5	92.0	92.1	92.9	90.5	93.2	90.0	92.0	92.0
	Livestock shows	84.7	79.5	**85.1**	**78.7**	**84.8**	**78.8**	**85.0**	**78.4**	**84.5**	**78.6**
	Pets	90.4	93.2	90.3	93.3	90.9	92.5	92.0	90.6	91.3	92.0
	Petting zoo	80.0	76.5	79.1	77.7	78.2	79.1	80.0	76.1	79.1	77.5
	Police animals	88.1	88.4	88.5	87.8	88.4	87.9	89.3	86.5	88.8	87.0
	Therapy animals	91.4	91.7	91.1	92.1	91.1	92.2	92.8	89.4	91.1	92.4
	Hunting	**67.9**	**55.7**	**67.8**	**55.5**	**66.7**	**56.5**	**67.6**	**55.2**	**66.3**	**56.2**
	Horse racing	**70.3**	**59.2**	**70.2**	**59.1**	**69.9**	**58.8**	**70.1**	**58.7**	**69.0**	**59.4**
	Dog racing	**57.5**	**42.9**	**57.1**	**43.0**	**56.5**	**43.1**	**57.7**	**41.3**	**56.1**	**42.4**
	Horse-drawn carriage	**82.0**	**69.6**	**82.9**	**68.0**	**81.5**	**69.3**	**81.6**	**69.4**	**80.1**	**70.7**
	Zoo animals	**79.8**	**69.3**	**79.3**	**69.8**	**78.4**	**70.6**	**79.4**	**69.0**	**78.3**	**69.9**
	Circus animals	**58.7**	**43.2**	**57.7**	**44.2**	**56.8**	**44.8**	**57.9**	**43.2**	87.8	89.1

Note 1: In the interest of brevity, the values representing the percentage of individuals in each category who found the animal use unacceptable has been omitted. Because the values in each column must necessarily total 100, omitted variables can be calculated. Note 2: The numbers appear in bold when there was a statistically significant difference at the 5% level. Note 3: For the purposes of this table, (1) extremely concerned and (2) relatively concerned were aggregated into a single category being concerned about the species in question.

## References

[1] Driscoll J.W. (1992). Attitudes toward animal use. Anthrozoös.

[2] Maher B. Free Pigs from the Abusive Crates, 2014. http//www.nytimes.com/2014/10/18/opinion/free-pigs-from-the-abusive-crates.html?_r=0.

[3] Kroger Kroger Announces Goal of 100% Cage-Free Eggs by 2025, 2016. http//www.thekrogerco.com/docs/statements-policies/kroger-statement-on-cage-free-eggs.pdf.

[4] Baker B. Maine Puts Bear Baiting on the Nov. Ballot, 2014. http//www.bostonglobe.com/metro/2014/08/30/maine-bear-hunt-challenged-ballot/oPbtDqPxNBxtUfgB8eMZkI/story.html.

[5] Perry T. Amid “Blackfish” Backlash, SeaWorld to Expand Orca Environments, 2014. http//www.latimes.com/local/lanow/la-me-ln-seaworld-orca-plans-20140814-story.html.

[6] Allen G. SeaWorld Agrees to End Captive Breeding of Killer Whales, 2016. http//www.npr.org/sections/thetwo-way/2016/03/17/470720804/seaworld-agrees-to-end-captive-breeding-of-killer-whales.

[7] Mettler K. After 145 years, Ringling Bros. Circus Elephants Perform for the Last Time, 2016. https://www.washingtonpost.com/news/morning-mix/wp/2016/05/02/after-145-years-ringling-bros-circus-elephants-perform-for-the-last-time/?utm_term=.63e59c54c942.

[8] New York Times (2015). Why Not Retire the Circus Elephants Now?. http//www.nytimes.com/2015/03/07/opinion/why-not-retire-the-circus-elephants-now.html?_r=0.

[9] Jabs J., Devine C.M., Sobal J. (1998). Model of the process of adopting vegetarian diets: Health vegetarians and ethical vegetarians. J. Nutr. Educ..

[10] McKendree M.G.S., Croney C.C., Widmar N.J.O. (2014). Effects of demographic factors and information sources on United States consumer perceptions of animal welfare. J. Anim. Sci..

[11] McKendree M.S., Croney C.C., Widmar N.O. (2014). Bioethics Symposium II: Current factors influencing perceptions of animals and their welfare. J. Anim. Sci..

[12] Cummins A.M., Widmar N.J.O., Croney C.C., Fulton J.R. (2016). Exploring agritourism experience and perceptions of pork production. Agric. Sci..

[13] Serpell J.A. (2004). Factors influencing human attitudes to animals and their welfare. Anim. Welf..

[14] Wells D.L., Hepper P.G. (1997). Pet ownership and adults’ views on the use of animals. Soc. Anim..

[15] U.S. Census Bureau, 2014 State and Country Quick Facts. http://quickfacts.census.gov/qfd/states/00000.html.

[16] SAS Statistical Computations. SAS Procedures, 1999. http://v8doc.sas.com/sashtml/proc/zompmeth.htm.

[17] Wooldridge J.M. (2013). Introductory Econometrics: A Modern Approach.

[18] Vaske J.J., Jacobs M.H., Sijtsma M.T., Beaman J. (2011). Can weighting compensate for sampling issues in internet surveys?. Hum. Dimens. Wildl..

[19] Olsen S.B. (2009). Choosing between internet and mail survey modes for choice experiment surveys considering non-market goods. Environ. Resour. Econ..

[20] Knight S., Vrij A., Cherryman J., Nunkoosing K. (2004). Attitudes towards animal use and belief in animal mind. Anthrozoös.

[21] Grey2K Take Action: State by State, 2016. http//www.grey2kusa.org/action/states.html.

[22] Reade L.S., Waran N.K. (1996). The modern zoo: How do people perceive zoo animals?. Appl. Anim. Behav. Sci..

[23] Driscoll J.W. (1995). Attitudes toward animals: Species ratings. Soc. Anim..

[24] Phillips C., Izmirli S., Aldavood J., Alonso M., Choe B.I., Hanlon A., Handziska A., Illmann G., Keeling L., Kennedy M. (2010). An international comparison of female and male students’ attitudes to the use of animals. Animals.

[25] American Egg Board About the U.S. Egg Industry, 2016. http://www.aeb.org/farmers-and-marketers/industry-overview.

[26] Economic Research Service Milk Cows and Production by State and Region (Annual), 2016. http//www.ers.usda.gov/data-products/dairy-data.aspx.

[27] Criss D., Moghe S. Deal May Keep Horse-Drawn Carriages in New York, 2016. http//www.cnn.com/2016/01/18/us/new-york-horse-carriage-deal/.

[28] U.S. Department of the Interior, U.S. Fish and Wildlife Service, and U.S. Department of Commerce, U.S. Census Bureau, 2014 2011 National Survey of Fishing, Hunting, and Wildlife-Associated Recreation. http//www.census.gov/prod/2012pubs/fhw11-nat.pdf.

[29] PETA The Issues, 2016. http//www.peta.org/issues/.

[30] Humane Society of the United States The HSUS: Driving Transformational Change for Animals since 1954, 2016. http//www.humanesociety.org/about/hsus-transformational-change.html?credit=web_id93480558.

[31] Cornish A., Raubenheimer D., McGreevy P. (2016). What we know about the public’s level of concern for farm animal welfare in food production in developed countries. Animals.

[32] Robertson J.C., Gallivan J., MacIntyre P.D. (2004). Sex differences in the antecedents of animal use attitudes. Anthrozoös.

